# Has Clinical and Epidemiological Varicella Burden Changed over Time in Children? Overview on Hospitalizations, Comorbidities and Costs from 2010 to 2017 in Italy

**DOI:** 10.3390/vaccines9121485

**Published:** 2021-12-15

**Authors:** Maria Francesca Piazza, Daniela Amicizia, Chiara Paganino, Francesca Marchini, Matteo Astengo, Federico Grammatico, Cecilia Trucchi, Paolo Romairone, Simona Simonetti, Camilla Sticchi, Filippo Ansaldi

**Affiliations:** 1 Regional Health Agency of Liguria (ALiSa), 16121 Genoa, Italy; daniela.amicizia@unige.it (D.A.); chiara.paganino@alisa.liguria.it (C.P.); francesca.marchini@alisa.liguria.it (F.M.); matteo.astengo@alisa.liguria.it (M.A.); cecilia.trucchi@alisa.liguria.it (C.T.); camilla.Sticchi@alisa.liguria.it (C.S.); filippo.ansaldi@alisa.liguria.it (F.A.); 2 Department of Health Sciences (DiSSal), University of Genoa, 16132 Genoa, Italy; federico.grammatico@alisa.liguria.it; 3 Liguria Digitale S.p.A., 16121 Genoa, Italy; p.romairone@liguriadigitale.it (P.R.); s.simonetti@liguriadigitale.it (S.S.)

**Keywords:** varicella disease, epidemiological burden, economic burden, hospitalization, children, comorbidities, vaccination coverage, outpatient services, pharmaceutical costs

## Abstract

According to WHO estimates, varicella disease is responsible of a worldwide significant burden in terms of hospitalizations, complications, and deaths, with more than 90% of cases under 12 years old. This study aims at evaluating the clinical, epidemiological, and economic burden of varicella in Ligurian children, about comorbidities, organizational variables, and vaccination coverages from 2010 to 2017, in terms of Emergency Department accesses and hospitalizations. The overall hospitalization rate was 179.76 (per 100,000 inhab.), with a gradual but significant decline since 2015, when universal varicella vaccination was introduced in Liguria (*p* < 0.0001). The risk of being hospitalized for complicated varicella in subjects with at least one comorbidity was significantly higher than in subjects without comorbidities (*p* = 0.0016). The economic analysis showed higher costs in subjects with complicated varicella who were 0–3 years old. This age group showed higher costs also considering extra-hospital costs for both outpatient procedures and pharmaceutical costs (*p* < 0.0001). The results confirm the relevant burden of varicella, especially in the 0–3 age group and in children with comorbidities. Thus, vaccination with the achievement of adequate vaccination coverages is confirmed to be a necessary control strategy to reduce hospitalizations and associated complications with important economic benefits.

## 1. Introduction

The varicella-zoster virus (VZV) is a DNA virus of the herpes virus family. The primary infection leads to varicella disease; viral reactivation can cause herpes zoster [1,2,3].

Nowadays, varicella is a vaccine-preventable infectious disease. According to the World Health Organization (WHO), the annual worldwide burden of varicella is estimated to be approximately 140 million cases with 4,200,000 severe complications that require hospitalization, and 4200 deaths [4]. The available epidemiological data report that 52–78% of the cases occur in children aged six years or under, and 89–95.9% of the cases occur before adolescence (i.e., under 12 years of age) [5,6]. It is known that 2–6% of varicella cases attending a pediatrician/medical practitioner develop complications.

While usually varicella is a mild or moderate disease [6,7,8,9], a small proportion of varicella cases are severe with complications such as superinfections of the skin and soft tissue, respiratory syndromes, and neurological manifestations [5,6]. Long-term sequelae have been reported in 0.4% to 3.1% of hospitalized patients. The risk of developing serious varicella, or death, is higher in young children, the elderly, immunocompromised subjects, and individuals with underlying conditions. However, the susceptibility of healthy children to most complications, hospitalizations, and deaths caused by varicella emphasizes the importance of recognizing varicella as a health priority [6,10,11,12,13,14,15].

As a result of the healthcare and economic burden of varicella, many countries have introduced immunization in infancy. In particular, the varicella vaccine was firstly produced in Japan in the 1970s but then introduced in most western countries, including Germany, Sweden, Korea, the United States, and Italy in the late 1980s and 1990s [16]. Nowadays, two types of vaccines against varicella are available: monovalent varicella and quadrivalent Measles-Mumps-Rubella-Varicella. All vaccines against varicella are effective and safe in protecting against VZV infection and in reducing the severity of the disease [1,17] and elicit high seroconversion rates (about 90% and 99% of immunized children develop virus-specific protective antibody titers after the first and second dose, respectively) [4].

Furthermore, vaccination against varicella reduces the number, size, and duration of varicella outbreaks [6,18] and rapidly reduces disease incidence over time [15], thereby reducing societal and economic burden [15]. Despite these benefits, there is no consensus across Europe on immunization policy. As of 2014, in Europe varicella vaccine recommendations were heterogeneous; universal childhood vaccination was recommended at the national level only in five countries (Cyprus, Germany, Greece, Latvia, and Luxembourg), and at the regional level in Spain and in Italy [19]. Despite having adopted different varicella vaccination policies, most Italian Regions gradually introduced the childhood varicella vaccine into their immunization programs starting in 2003 and, after the vaccine introduction, the overall incidence decreased from 6.7 per 1000 population in 2003 to 1.7 per 1000 population in 2012 [20,21].

In Liguria, a Northern Italian Region, the varicella universal vaccination was introduced in 2015 for the 2014 birth cohort following the National Recommendation [22]. The Italian National Vaccination Plan 2017–2019, extended to 2021, recommends the administration of a first dose at 13–15 months of age and a second dose at 5–6 years of age. A vaccine coverage rate of ≥95% and two doses schedule should be achieved and maintained according to the Italian National Vaccination Plan [23].

The European Centre for Disease Prevention and Control [ECDC] recommends implementing epidemiological surveillance in order to monitor both the spreading of the pathogen and the impact of already implemented vaccine strategies [6]. As for the specificity of the implemented vaccination strategies in Italian Regions and the obtained suboptimal coverage rate, it is of interest to describe the burden of varicella during the time.

This study aims to estimate the clinical, epidemiological, and economic varicella burden in children in Liguria.

## 2. Materials and Methods

### 2.1. Study Design

A retrospective cohort study was performed in order to evaluate the clinical, epidemiological, and economic burden of varicella in terms of emergency department (ED) accesses and/or hospitalization from 2010 to 2017 in Liguria. The burden was then evaluated according to vaccination coverage, organizational variables, and comorbidities.

### 2.2. Study Population and Study Period

ED accesses/hospitalizations for varicella from January 2010 to December 2017 in Ligurian children (birth cohorts from 2000 to 2017, range 9872–12,062 newborns) were analyzed. Patients were captured by means of the ED and hospitalizations discharge records (ICD-9-CM 052* codes in any field of diagnosis) and the regional Chronic Condition Data Warehouse (CCDWH).

### 2.3. Data Sources

Different data sources were analyzed: (i) ED and hospital discharge records for the estimation of ED access/hospitalization of subjects with varicella; (ii) for Regional pediatric population: vaccine coverage using the regional vaccine registry, distribution by age group and presence of comorbidities obtained through the regional CCDWH; (iii) hospital pathways through ED and hospital discharge record; (iv) hospital and extra-hospital costs through the Regional Data Warehouse (hospital discharge record, pharmaceutical consumption, and outpatient specialist).

#### 2.3.1. Hospital Discharge Records

The ED discharge record is included among the administrative healthcare data routinely collected. At the end of each ED access, ED discharge records periodically were sent to the Regional Authority. The ED discharge records include various data such as the main diagnosis field coded by the ICD-9-CM system, the procedures and treatments administered during the ED access, the way of admission and discharge, and the severity of the condition at the access time. Administrative healthcare data such as ED discharge records display weaknesses in terms of sensitivity and specificity. Incidence rates and other variables were defined by using data extracted from this registry, selecting all hospital admissions with a main diagnosis of varicella or its complications (International Classification of Diseases, Ninth Revision (ICD-9-CM) codes. (Table 1).

#### 2.3.2. Chronic Condition Data Warehouse (CCDWH)

The CCDWH records data were gathered from multiple Medicare data sources (hospital discharge records, pharmaceutics, medical fee exemptions, outpatient visits, and laboratory/imaging procedures) within a specified period using a predefined algorithm based on the codes assigned to specific diagnoses and procedures. Through a record-linkage system based on a civil registry database, residents’ histories of healthcare events are constructed to depict the chronic condition of each patient. All data are archived in a relational database (RDBMS) using big-data logic. The main diseases recorded by the CCDWH are the following: organ transplantation, renal failure, HIV infection, malignancies, diabetes, cardiovascular diseases, bronchopneumopathies, gastrointestinal diseases, neuropathies, autoimmune diseases, endocrine metabolic disorders, and rare diseases. A unique code specific for each patient is used to link data from our study population and the CCDWH and to evaluate the patient’s chronic conditions on the day of ED access. To calculate crude incidence by health care setting, pediatric demographic data were extracted from the regional Chronic Condition Data Warehouse (CCDWH).

#### 2.3.3. Varicella Vaccination Offer

In the Liguria region, the varicella vaccine was recommended only for subjects at risk, as identified by the National Vaccine Prevention Plan 2003–2005, and administered with an active offer in healthy and susceptible adolescent subjects (one dose in 11–12-year-old with negative anamnestic criterion). Subsequently, according to the 2015 Regional Vaccination Prevention Plan (Regional Council Deliberation no. 1701 del 22 December 2014), the Ligurian immunization policies included a universal varicella vaccination program with an active and free-of-charge immunization strategy addresses to all children aged 15 months old (beginning from 2014 birth cohort), with a second dose administered at 5–6 years of age. A catch-up two-doses vaccination was also offered free of charge to 12-year-old adolescents with a negative history of varicella. According to the present recommendations, the coverage target was set at ≥95% coverage.

In order to monitor the coverage rate, aggregated regional data collection for administrative purposes were used; provided by the local health authorities. In the present study data on vaccination coverage were retrieved from this database.

### 2.4. Direct Costs

Cost estimates of ED accesses, including admission to short observation, and the direct costs of hospitalizations were obtained by scrutinizing the outpatient procedures performed in the ED and the Diagnosis-related group (DRG) system, respectively. By the regional reimbursement system, the evaluation of costs related to ED accesses followed by hospitalization included only the DRG-based costs. Extra-hospital costs (pharmaceutical and outpatient specialist related to the diagnosis and treatment of varicella occurred 6 months before and 6 months after the ED access for febrile vesicular rash) were obtained through the Regional CCDWH Data Warehouse.

### 2.5. Statistical Analysis

A descriptive analysis of the pediatric varicella ED accesses and hospitalizations was performed. Results were expressed as a median and interquartile range for quantitative outcome measures and as frequency distributions for qualitative outcome measures. Rates were estimated together with the corresponding 95% confidence intervals (CIs).

Different statistical association tests were used depending on the type of outcome measure: to compare qualitative and quantitative variables, the χ^2^ test (Pearson test) and Mann–Whitney U test, were applied respectively. Comparison of outpatient procedures and pharmaceutical costs at the 6 months before and after ED access/hospitalization was carried out using the Wilcoxon test. Data were analyzed by JMP version 13.0.0 software (SAS Institute, Cary, NC, USA). A value of *p* < 0.05 was considered statistically significant.

## 3. Results

### 3.1. Epidemiological and Clinical Burden

A total of 2239 ED accesses and/or hospitalizations for varicella among Ligurian children 0 to 17 years were reported from 2010 to 2017, of which 2014 (89.95%) ED accesses and 225 (10.05%) hospitalizations were preceded or not by ED access.

Regarding demographic characteristics, the overall median age of varicella cases was 4 years (IQR 2–6) and 3 years (IQR 1–5) considering hospitalizations preceded or not by ED access respectively. Over the entire period, 52.88% of cases were observed in males without statistically significant difference between gender (*p* = 0.145).

The Liguria region includes five LHAs (Local Health Authorities), covering the entire regional population. The highest incidence was observed in LHA2 (240.13 per 100,000 inhabitants), followed by LHA1 (206.27 per 100,000 inhabitants) considering the total events and ED accesses; instead for hospitalization preceded or not by ED access the highest incidence was observed in LHA3 (23.62), followed by LHA2 (17.25).

**Figure 1:**
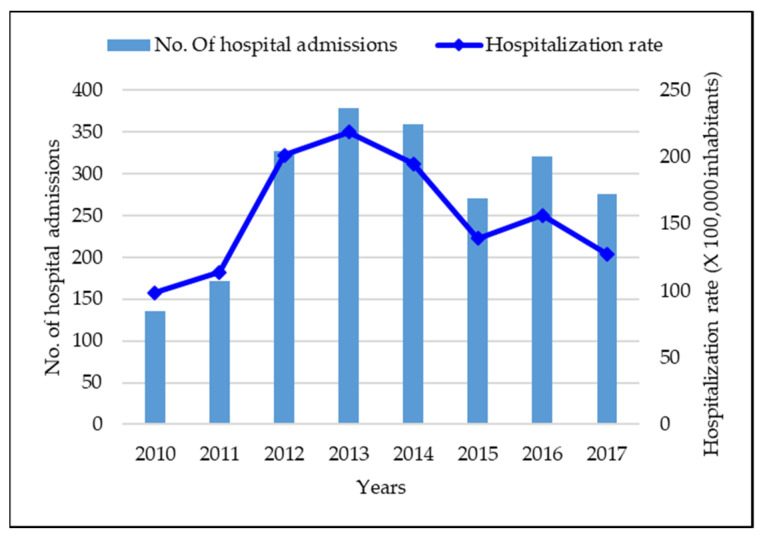
Number of hospitalizations due to varicella and hospitalization rate per 100,000 inhabitants in the Liguria region in Ligurian children aged 0–17 years old.

**Figure 2:**
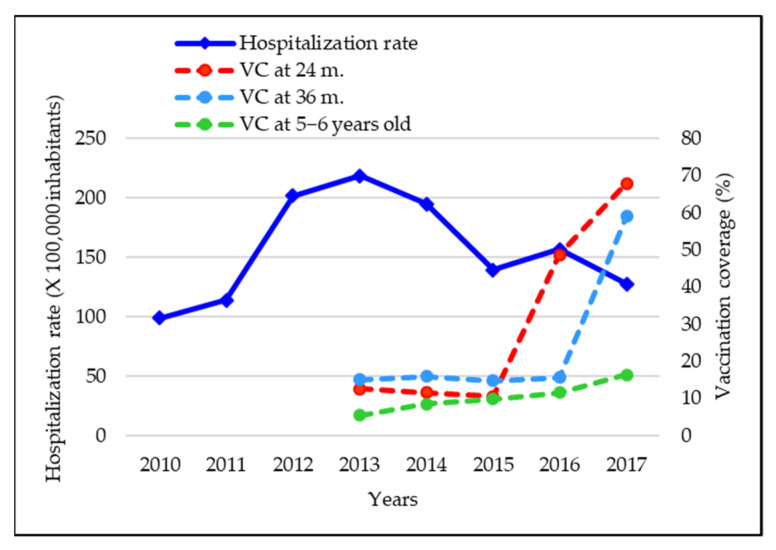
Varicella ED accesses/hospitalizations per 100,000 inhabitants and vaccination coverage (VC) in Liguria at 24, 36 months and 5–6 years of age.

Overall, the total incidence of varicella cases in our study population was 179.76 per 100,000 inhabitants. In particular, the total incidence rate of ED accesses and hospitalizations preceded or not by ED access for varicella in children was equal to 161.70 and 18.06 per 100,000 subjects, respectively. The annual incidence rate of cases showed an increasing trend between 2010 and 2012, reached a peak in 2013, with an incidence of 218.58 per 100,000 inhabitants, and from 2015 it declined, reaching 127.46 per 100,000 population in 2017 (Figure 1), in particular according to the upward trend of vaccine coverages at 24 months of age for single antigen that reached values of 10.57% in 2015 (birth cohort 2013), 48.74% in 2016 (birth cohort 2014), 67.89% in 2017 (birth cohort 2015) (Figure 2).

The vaccination coverages at 36 months and 5–6 years of age showed very low values in 2015 and 2016 and higher values starting from 2017, thus we did not consider these coverages associated with the declining trend of hospitalization rates in our study period. Two further clarifications need to be made considering the annual hospitalization rates (Figure 1). In fact, lower rates in 2010 and 2011 and a rebound in 2016 were observed.

The first aspect may be explained by an underestimation of varicella cases due to a lack of accuracy of the electronic administrative regional flows in Liguria Region that was reinforced starting from 2010.

While the rebound in 2016 may be related to the technical timing of implementation of the universal varicella vaccination program addressed to all children aged 15 months old. This document was published in January 2015, but implemented in the subsequent months, thus, there may have been a delay in the visibility of the most effects of vaccination.

**Figure 3:**
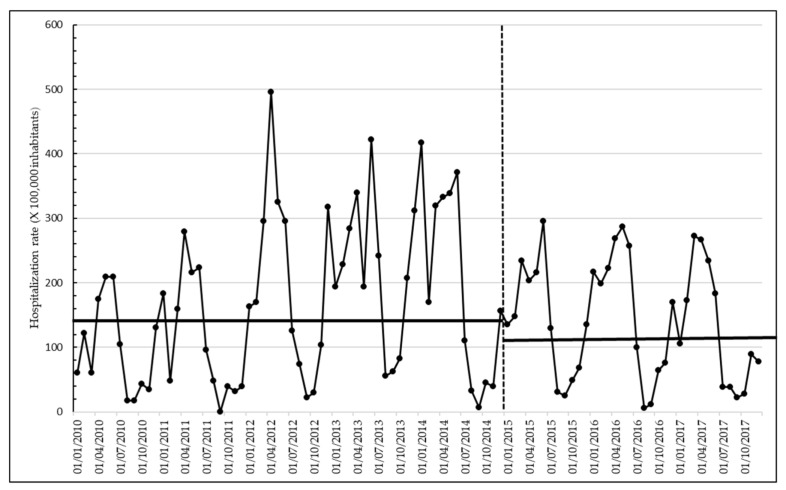
Monthly hospitalization rates of varicella in Ligurian subjects aged 0–17 years old, study period 2010–2017.

More details on downward trend hospitalization rates, in a transition period between immunization policies, were more evident in Figure 3, that splitting data in a monthly hospitalization rate, showed more clearly the little differences and the declining trend from 2015 to 2017.

In particular, data showed a seasonal pattern of the epidemiology of varicella with a peak incidence during spring months (from February to May) and a lower incidence in late summer and early autumn. During the years January 2010–December 2014, a period in which vaccination had been already introduced, but coverage was still very low, this seasonal trend continued, however, to level up. The analysis identified the breakpoint as occurring in January 2015, in which peak incidence rates decreased significantly (*p* < 0.0001, OR 1.532, 95% CI 1.225, 1.917). The average varicella incidence rate among 0–17 year-olds from January 2010 to December 2014 (before the breakpoint) was 169.83 per 100,000 person-years. Average incidence decreased to 140.84 after January 2015. The yearly peaks in varicella hospitalization rates decreased after the vaccine was introduced and then fell further as higher coverage were achieved, as previously reported in Figure 1 (Figure 3).

An increasing trend of ED accesses/hospitalizations for varicella disease was generally observed from 2010 to 2014 in children aged 0–3 years old and from 2010 to 2013 in the other age groups. A decreasing trend was observed from 2015 to 2017 in children aged 0–3 years old and from 2014 to 2017 in the other age groups.

In our study population, 2099 cases with a primary diagnosis of varicella (median age 4, 25–75p 2–6) and 140 cases with secondary diagnosis (median age 3, 25–75p 2–5) were recorded. Most of the patients (2083; 93.03%) was healthy, while 6.97% (156) had at least one comorbidity, of which 121 (6.01%) required ED access and 35 (22.43%) required hospitalization preceded or not by ED access.

Among cases with comorbidities, the most common were bronchopneumopathies (87/156, 55.77%) followed by cardiovascular diseases (32/156, 20.51%). Asthma (98.85%) was the most frequent bronchopneumopathy, while valvular heart diseases (84.38%) were the most frequent among cardiovascular diseases (Table 2).

In the comparison between comorbidities, it was observed that broncopneumopathy was the comorbidity most implicated in ED access/hospitalization for varicella than the other comorbidities (*p* = 0.0014, OR 1.629, 95% CI 1.204, 2.204) (Table 3).

Overall, ED accesses and/or hospitalization with a diagnosis of complicated varicella infection were 192 (8.58%). The risk to be hospitalized for complicated varicella in subjects with at least one comorbidity (24/132, 18.18%) was significantly higher than subjects without comorbidities (168/1915, 8.77%) (*p* = 0.00164, OR 2.072, 95% CI 1.282, 3.254).

Among the complications reported in the hospital discharge records, 8.33% (16/192) of the hospitalizations were due to post-varicella encephalitis, 0.52% (1/192) to varicella-linked hemorrhagic pneumonia, 0.52% (1/192) to post-varicella myelitis, 67.71% (130/192) to varicella with other complications, 22.92% (44/192) with complications not specified.

Regarding the hospital pathways followed by patients, the admission took place mainly in directly managed public hospitals through emergency department access. Specifically, 223 were hospitalized in ordinary regimen, while only two patients were hospitalized in day-hospital regimen.

Children were mainly hospitalized in Gaslini Institute, one of the major pediatric hospital in Italy, located in Genoa, that cared for 67.56% of cases. The overall median length of hospital stay was four days (min = 0; max = 42 IQR 3–6). It did not change over the seven-year period (*p* = 0.2909). The median length of hospital stay increases with age. In particular, in the population 0–3 years the median length was four days (IQR 3–6), 4–7 years was four days (IQR 3–7), and for children ≥8 it was four and a half days (IQR 2.75–7.25, *p* = 0.5274). Infectious diseases represented the most frequent admission, transfer, and discharge ward, followed by emergency medicine. The main way of discharge was at the patient’s home (96.44%).

### 3.2. Economic Burden

Estimates of the economic burden of varicella in terms of ED accesses/hospitalizations showed a total cost of €624,305 of which €45,945 was for ED accesses and €578,360 was for hospitalization preceded or not by ED access in the study period 2010–2017. Considering the total costs adjusted for inflation on December 2017, ED accesses/hospitalizations for varicella amounted to €642,634, of which €46,984 was about ED accesses and €595,649 for hospitalization preceded or not by ED access (Table 4).

The overall cost for a single event amounted to €273, of which €23 was about ED accesses. Considering the total costs adjusted for inflation on December 2017, ED accesses/hospitalizations for varicella amounted to €281, of which €23 was about ED accesses. On stratification by age group, the total costs varied from €348,979 in children 0–3 years old (per-capita cost €347), €206,169 in 4–7 years old (per-capita cost €215), and €69,157 in ≥ 8 years old (per-capita cost €254).

The total cost of ED access/hospitalization in patients with complicated varicella was equal to €385,889 versus €238,416 (cost of varicella without complication).

The total cost of ED access/hospitalization stratified by age-groups revealed the highest costs in the 0–3 age group (€193,364 vs. €155,614) than the other age groups (€192,525 vs. €82,802), both for varicella with and without complications.

Outpatient procedure and pharmaceutical costs in the six months before and after ED access/hospitalization stratified by age in Ligurian subjects aged 0–17 years old were analyzed. In particular, children of 0 years old showed higher cost in the six months after ED access/hospitalization vs. the six months before, both for outpatient procedures (+€1951) and pharmaceutical costs (+€525), children aged 1, 2, 9 years showed higher costs in the six months after ED access/hospitalization vs. the 6 months before for pharmaceutical costs (+€296, +€1565, +€1135, respectively), and, finally, children aged 3 and 5 years showed higher costs in the six months after ED access/hospitalization vs. the six months before for outpatient procedures (+€4855 and +€1380).

From a comparison between the 6 months after and before ED, access/hospitalization related to extra-hospital costs, the age group 0–3 years showed costs significantly higher than the other ages in the six months after ED access/hospitalization both for outpatients procedures and pharmaceutical consumption (*p* < 0.0001, OR 1.308, 95% CI 1.285, 1.331; *p* < 0.0001, OR 1.15, 95% CI 1.121, 1.18, respectively).

### 3.3. Vaccination Coverages in the Study Population

Among 2239 children hospitalized, 101 (4.51%) received at least one dose of varicella vaccine. Specifically, 44 children received the first dose at least one month before the ED access/hospitalization, 48 children after the event, and none subjects in a range from zero to 15 days before the ED access/hospitalization. In particular, eight children required ED access, and one child required hospitalization preceded by ED access developing encephalitis post varicella resolved within 16 days of hospitalization discharged at home.

Among 44 children who received the first dose at least one month before the ED access/hospitalization, a second dose was administered to 15 children (12 before the ED access/hospitalization and three after).

Furthermore, among 48 children who received the first dose after the ED access/hospitalization, a second dose was administered to 4 children.

## 4. Discussion

WHO advocates routine varicella immunization in countries where high vaccination coverage can be achieved (since low coverage could potentially increase the incidence of varicella cases in older children and adults, increasing the burden of disease) [4].

Furthermore, As recommended by the ECDC, the assessment of epidemiological variations linked to the implementation of vaccine strategies is mandatory to further improve national programs [6].

In Italy, during the last decade, regional recommendations on varicella vaccination were heterogeneous and led to a marked discrepancy in vaccination coverage observed throughout the country. In particular, a 1-dose varicella vaccination program was introduced in Sicily [24] and Apulia in 2003 and 2006, respectively, for all children aged approximately 15 months and for all susceptible adolescents at 12 years of age in Sicily. By 2011, eight regions (Sicily, Apulia, Basilicata, Calabria, Friuli-Venezia Giulia, Sardinia, Tuscany, and Veneto) adopted a universal varicella vaccination program targeting children and susceptible adolescents, whilst in the remaining 13 regions, varicella vaccination had been offered free of charge only to risk groups, susceptible adolescents and susceptible women of childbearing age.

Additionally, an Interregional Group on Varicella Vaccination has been established in 2013 to assess the effectiveness of the immunization programs started in these regions, providing a standardized method to collect data.

In Liguria, the varicella universal vaccination was introduced in 2015 for the 2014 birth cohort following the National Recommendation [22]. According to the 2015 Ligurian Vaccination Prevention Plan, the immunization policies included a universal varicella vaccination program with an active and free-of-charge immunization strategy addressed to all children aged 15 months old (beginning from 2014 birth cohort) with a second dose administered at 5–6 years of age. A catch-up two-doses vaccination was also offered free of charge to 12-year-old adolescents with a negative history of varicella [25]. According to these recommendations, the coverage target was set at ≥95% coverage.

This actual immunization scenario is in line with the results of the study that showed a gradual decline of hospitalization rate of ED access/hospitalization for varicella in patients aged 0–17 years in Liguria, starting from 2015. This could be implied with the beginning of the varicella vaccination strategy. Our findings highlight that the breakpoint occurred in January 2015, when peak incidence rates decreased. As previously described, this could be linked to prevention measures adopted. Nevertheless, observing in detail the annual incidence rate of cases (Figure 1) there were lower rates in 2010 and 2011 and a rebound in 2016. The lower rates in 2010 and 2011 may be explained by an underestimation of varicella cases due to a lack of accuracy of the electronic administrative regional flows in Liguria Region that was reinforced starting from 2010.

The rebound in 2016 could be related to the Ligurian Vaccination Prevention Plan that included a universal varicella vaccination program with an active and free-of-charge immunization strategy addressed to all children aged 15 months old, which was published on January 2015 but implemented subsequently. Thus, it is supposed that the most important effects of the immunization program on hospitalization rates were visible starting from 2016, when higher vaccination coverages were reached. In fact, as well as confirmed in several studies hospitalization rates are strictly related to both the vaccination coverage and the number of years since the introduction of the vaccination [21,26,27,28].

In particular, Figure 1, which reports data using the “year” as the unit of measure, has not the objective to show a trend or the minimal differences of hospitalization rates between years, but it gives an idea of the global epidemiological burden over time in Liguria Region. On the other hand, more details on the hospitalization rates trend, in a transition period between immunization policies, were more evident in Figure 3, whereby splitting data in a monthly rate showed more clearly the little differences and the declining trend.

In this scenario, varicella vaccination is confirmed to be an important step in public health strategies and the introduction of universal vaccination, with high vaccination coverage, should be considered as an extremely powerful tool to reduce the risk of complications.

Furthermore, it was found that the risk to be hospitalized for complicated varicella in subjects with at least one comorbidity was significantly higher than subjects without comorbidities. The comparison between comorbidities revealed that broncopneumopathy and cardiovascular disease were the comorbidities most implicated in ED access/hospitalization for varicella than the other comorbidities; however, statistical significance was reached only for broncopneumopathy. Others studies have found analogous results [29,30].

Estimates of the economic burden of varicella in terms of ED accesses/hospitalizations showed lower costs with respect to previous not recent Italian published data, [31,32]; it is linked to the fact that the majority of cases required only ED access.

We found higher costs in children 0–3 years old both for varicella with and without complications than the other age-groups. Similarly, as regards extra hospital costs, the age group 0–3 years showed costs significantly higher than the other ages in the six months after ED access/hospitalization, both for outpatients procedures and pharmaceutical costs.

An increase in vaccination coverage rate was detected starting from 2015; however, a suboptimal coverage rate was observed in 2016 and 2017, far from the threshold recommended by the Italian Minister of Health [≥95%].

The vaccination coverages, suboptimal in the general population, were also unsatisfactory in hospitalized patients. Specifically, 44 children received the first dose at least one month before the ED access/hospitalization, showing a vaccination failure of 1.95%. Other authors found higher percentages in similar populations: Kuter et al. and Prymula et al. reported 4% and 5%, respectively [33,34].

These observations, derived from our results, imply that varicella disease has a relevant impact on public health in terms of hospitalizations, complications, and costs, especially in early childhood. Furthermore, the costs related to hospitalizations for complicated varicella and extra-hospital costs (outpatient procedures and pharmaceuticals costs) were also a considerable issue. Nevertheless, it is also worth considering indirect costs, not assessed in this study, relating, for example, to the number of lost working days by parents or family members for the care of children affected by varicella and also the indirect effects of the disease on the health of adults and elderly, often more at risk of severe effects of the disease. Thus, reaching higher vaccination coverages is necessary not only to prevent severe cases of varicella in children but also to avoid the risk of increased incidence of severe varicella in older age groups, where VZV infection can have more severe adverse consequences [35].

Thus, our experience supports the intention to reinforce the national universal routine vaccination against varicella in children and the routine assessment of the epidemiology and disease burden of varicella to better address the planning and evaluating varicella control strategies.

This study could have some limitations that have to be considered. Firstly, our data, based on a large administrative database, may pose concerns relating to miscoding and data reporting inaccuracies. We cannot exclude the presence of underreporting of ICD-9 CM codes suggestive for varicella complications, in particular in the pediatric population. It should be also considered that, in Italy, in a large majority of cases, reporting varicella complications have a trivial impact on economic reimbursement and thus could be omitted by clinicians. Nevertheless, we are confident that, if errors occurred, they were similar throughout the whole study period. Furthermore, this study reports only severe cases requiring hospitalization, not considering mild cases, so, unfortunately, our data cannot be considered conclusive as the number of cases might be too low in general to provide robust results.

Despite these potential limitations, a strength of the present study is that we investigated varicella-related hospitalizations through data collected from all over the Liguria Region (population 1.56 million). The CCDWH platform consists of a sophisticated system that integrates several administrative regional flows and it is used to identify comorbidities with accuracy and precision. Furthermore, to the best of our knowledge, few studies in the world evaluated varicella-related hospitalization rates over a long temporal period, comparing these rates with varicella vaccine coverage. In this scenario, this study adds updated and suggestive findings to the international literature and shows that varicella hospitalizations in Italy, from 2010 to 2017, are an important healthcare burden, especially during years before varicella vaccination.

## 5. Conclusions

Our study demonstrates that varicella causes a substantial burden especially on early children and in children with comorbidities, better defining the picture of the real burden of this vaccine-preventable disease. These data support the strengthening of varicella vaccination programs in the infancy and high-risk children. The continuous updating of epidemiological data in terms of disease impact (hospitalizations, complications and costs) is crucial for the overall assessment of the impact of vaccination against varicella.

## Figures and Tables

**Table 1:** ICD-9-CM codes were used to select subjects with varicella.

ICD-9-CM	Description
052.0	Post-varicella encephalitis
052.1	Varicella (hemorrhagic) pneumonia
052.2	Post-varicella myelitis
052.7	Varicella with other specified complications
052.8	Varicella with unspecified complication
052.9	Varicella without mention of complication

**Table 2:** Comorbidities in Ligurian children aged 0–17 years old with an ED access/hospitalization for varicella, study period 2010–2017.

Comorbidities n. (%)	Total Events	ED Accesses	Hospitalization Preceded or Not by ED Access
Patients without comorbidities	2083 (93.03)	1893 (93.99)	190 (84.44)
≥1 comorbidity	156 (6.97)	121 (6.01)	35 (15.56)
C02—Transplant	0 (0.0)	0 (0.00)	0 (0.00)
C03—Chronic renal failure	2 (1.28)	0 (0.00)	2 (5.71)
C04—HIV/AIDS	0 (0.0)	0 (0.00)	0 (0.00)
C05—Cancer	4 (2.56)	2 (1.65)	2 (5.71)
C06—Diabetes	6 (3.85)	3 (2.48)	3 (8.57)
C07—Cardiovascular disease	32 (20.51)	26 (21.49)	6 (17.14)
C07S1—Hypertension	1 (3.13)	0 (0.00)	1 (16.67)
C07S2—Ischemic heart disease	0 (0.00)	0 (0.00)	0 (0.00)
C07S3—Valvular heart disease	27 (84.38)	23 (88.46)	4 (66.67)
C07S4—Arrhythmic myocardiopathy	3 (9.38)	1 (3.85)	2 (33.33)
C07S5—Non-arrhythmic myocardiopathy	0 (0.00)	0 (0.00)	0 (0.00)
C07S6—Heart failure	0 (0.00)	0 (0.00)	0 (0.00)
C07V1—Arterial vasculopathy	0 (0.00)	0 (0.00)	0 (0.00)
C07V2—Venous vascular disease	1 (3.13)	1 (3.85)	0 (0.00)
C07V3—Cerebral vasculopathy	3 (9.38)	2 (7.69)	1 (16.67)
C08—Bronchopneumopathy	87 (55.77)	69 (57.02)	18 (51.43)
C08A—Asthma	86 (98.85)	69 (100.00)	17 (94.44)
C08B—COPD	1 (1.15)	0 (0.00)	1 (5.56)
C08C—Respiratory failure/oxygen therapy	1 (1.15)	0 (0.00)	1 (5.56)
C09—Gastroenteropathy	3 (1.92)	2 (1.65)	1 (2.86)
C10—Neuropathy	13 (8.33)	9 (7.44)	4 (11.43)
C11—Autoimmune disease	3 (1.92)	2 (1.65)	1 (2.86)
C12—Endocrine and metabolic disease	5 (3.21)	2 (1.65)	3 (8.57)
C13—Rare disease	10 (6.41)	5 (4.13)	5 (14.29)
C14—Psychosis	6 (3.85)	6 (4.96)	0 (0.00)

A higher incidence of ED access/hospitalization for varicella was observed in patients with broncopneumopathy and cardiovascular disease.

**Table 3:** Comorbidities implicated in ED access/hospitalization for varicella.

Comorbidities	OR	Low 95% CI	Up 95% CI	*p*-Value
C02—Transplant	-	-	-	-
C03—Chronic renal failure	2.488	0.294	9.409	0.397
C04—HIV/AIDS	-	-	-	-
C05—Cancer	0.391	0.105	1.022	0.057
C06—Diabetes	1.726	0.620	3.873	0.293
C07—Cardiovascular disease	1.178	0.790	1.718	0.405
C08—Bronchopneumopathy	1.629	1.204	2.204	0.001
C09—Gastroenteropathy	0.496	0.101	1.483	0.307
C10—Neuropathy	1.108	0.576	1.957	0.808
C11—Autoimmune disease	0.641	0.130	1.916	0.635
C12—Endocrine and metabolic disease	0.539	0.172	1.287	0.214
C13—Rare disease	0.442	0.208	0.836	0.008
C14—Psychosis	0.734	0.265	1.639	0.600

**Table 4:** Costs of ED accesses/hospitalizations for varicella (adjusted for inflation at December 2017), stratified by year and healthcare setting.

	Total Events		ED Accesses		Hospitalization Preceded or Not by ED Access	
Year	Number	Costs (Adjusted for Inflation)	Number	Costs (Adjusted for Inflation)	Number	Costs (Adjusted for Inflation)
2010	138	€61,747 (€67,489)	113	€2076 (€2269)	25	€59,670 (€65,219)
2011	173	€72,058 (€77,102)	135	€2549 (€2727)	38	€69,509 (€74,375)
2012	334	€66,037 (€68,480)	308	€6757 (€7007)	26	€59,280 (€61,473)
2013	390	€100,147 (€101,649)	351	€8049 (€8170)	39	€92,097 (€93,478)
2014	363	€71,941 (€72,588)	333	€7945 (€8017)	30	€63,996 (€64,573)
2015	271	€44,670 (€45,429)	257	€6287 (€6373)	14	€38,383 (€39,036)
2016	332	€127,852 (€129,642)	289	€6285 (€6373)	43	€121,567 (€123,269)
2017	285	€79,854 (€80,253)	255	€5997 (€6027)	30	€73,857 (€74,226)
Total	2286	€624,305 (€642,634)	2041	€45,945 (€46,984)	245	€578,360 (€595,649)

## Data Availability

The data are not publicly available due to privacy or ethical restrictions.
